# Foreign outsourcing collaboration within a developing economy’s perspective: A case of the Pakistani textile industry

**DOI:** 10.1371/journal.pone.0299454

**Published:** 2024-04-16

**Authors:** Irfan Ali, Zafar Mahmood

**Affiliations:** Department of Economics, School of Social Sciences and Humanities (S3H), National University of Sciences & Technology (NUST), Islamabad, Pakistan; Istinye University: Istinye Universitesi, TURKEY

## Abstract

This paper develops an outsourcing collaboration model from a firm’s perspective operating in a developing economy. The model considers that producers of the final goods residing in a developed country, and operators of manufacturing plants in a developing country collaborate with each other. The final goods producer supplies headquarter services for the production of intermediate goods in the developing country. The operators of manufacturing plants also supply their services in the domestic economy. This arrangement leads to foreign outsourcing collaborations (FOC) between firms of developed country and developing country. The operators of manufacturing plant maximize revenue subject to the cost constraint. The first order conditions suggests that an increase in wages of skilled labor, price of domestic inputs, and cost of production deter FOC. On the other hand, an increase in demand for and price of foreign headquarter services increases the FOC. Empirical analysis based on data collected from 217 clothing (textile and apparel) firms in the city of Faisalabad (Pakistan) reveals that an increase in wage to labor-productivity ratio reduces FOC. An improvement in skilled of the labor and foreign headquarter services give rise to FOC, whereas an increase in economies-of-scope enhances FOC. Additionally, an inverted U-shaped relationship is found between the cost of production and FOC, which shows that at the initial stage, the firm’s cost of production increases with an increase in the level of FOC, but soon after the tipping point, the firm’s cost starts decreasing with a further increase in FOC.

## 1. Introduction

The global trading environment is rapidly changing, with the shortening of product life cycles, emergence of new industrial patterns, shifting economic infrastructure requirements, and emerging patterns in the division of labor [1]. Producers are responding to these factors to gain competitive advantage, while laggards, or firms who do not adapt to innovations, permanently lose their export market shares. Industrial patterns are shifting from vertically integrated to network arranged and new arrangements of industrial concentration, local supplier relationships, outsourcing, and international distribution networks have increasingly become relevant in determining international trade patterns.

Pakistan has liberalized its economy since the early 1990s, encouraging foreign investors to come to Pakistan and invest in manufacturing industries, including textiles and apparel. Policies that allow foreign investors to acquire businesses through acquisition and merger to form solely owned firms and/or collaboration to establish a joint venture have boosted investors’ confidence. Foreign firms that are unable to come to Pakistan engage in outsourcing collaborations to take advantage of cheap labor, increasing their competitive advantage.

Foreign outsourcing of products and services is a well-known approach employed by businesses to obtain a competitive edge in the global supply chain (GSC). Firms based in developed nations engage in foreign outsourcing operations for a various reasons, including a reduction in the cost of production [2–4]. Such activities have been facilitated by trade liberalization, efficient transportation services, an improved trade facilitation system, and advancements in information and communication technology (ICT).

Foreign outsourcing has significantly benefited developing economy firms (DEFs), ensuing in the emergence of new enterprises, export revenues, job creation, and technological and skill up-gradation [5]. Such collaborations also allow DEFs to gain superior technology, R&D skills, new product development skills, and learning opportunities [6], providing a competitive advantage for enterprises in both developed and developing economies. These initiatives also aid in the development of new products and processes, and foster clusters since foreign outsourcers prefer clustered sites to individual enterprises due to cheaper labor and high-quality products and services. In contrast, vertically integrated firms face higher costs of production due to the lack of learning associated with specialization in a single activity, the absence of competition for specialization, and the additional cost associated with managing a large organization [7].

This study devlops a model identifying determinants of foreign outsourcing collaborations decisions from the perspective of DEFs while highlighting firm-specific characteristics affecting their decision. Specifically, this model assesses the role of wages of skilled labor, domestic and foreign headquarter services, and cost of production along with firm characteristics, efficiency, knowledge spillovers, agglomeration economies, etc. These factors have been incorporated in the model as they play a crucial role in foreign outsourcing collaboration decisions of DEFs. Such consideration enables us to focus their characteristics, identifying factors that boost their productivity, specialize in a single activity, and gain competitive advantage by focusing on the specific needs and requirements of final goods producers. Thus, by leveraging these benefits, DEFs can enhance their export share in the GSC and become a dominant player in the textile sector.

The data collected from the textile and apparel industry in the city of Faisalabad (Pakistan) have been used to empirically validate the model. Pakistan’s textile sector is the largest industrial group, accounting for 25% of the value-added of the manufacturing sector, 60% in total exports, and employing 40% of the total industrial labor force [8–10]. Despite being the largest manufacturing sector and having competitive advantage, Pakistan’s share of textile exports in world textile and clothing is fairly low i.e., 1.70% as compared to its main competitors Bangladesh (4.96%), Vietnam (4.79%), India (3.81%) [11]. Developed countries are using outsourcing of activities as a business strategy to enhance their competitiveness and increase their share of exports in total world exports. Countries like Pakistan are benefiting from sourcing collaboration activities by exploiting their potential and taking advantage from international fragmentation of production stages. Such benefits include human capital advantages, technological transfer and know-how, knowledge spillovers, agglomeration economies, production or social networks, labor pooling, and specialized suppliers.

The rest of the study is divided into 6 sections. Section 2 presents a detailed discussion of the literature. Section 3 introduces the theoretical model. Section 4 offers a comparative static analysis. Discussion on material, method and model is provided in section 5, while results and discussion are presented in section 6. Finally, section 7 concludes the paper and provide policy implications.

## 2. Literature review

The literature indicates the models that are being used determine the characteristics of firms involved in foreign outsourcing such models have been developed based on developed countries’ perspectives. The details of such models are presented in S1 Fig in S1 Appendix which provides an evolution of foreign outsourcing models including contracting, offshoring, insourcing, etc. Each box in the figure shows the name of the author(s) who developed a model of foreign outsourcing. The most prominent work in the development of outsourcing models has been carried out by Grossman & Helpman [7], Antras [12], Antràs & Helpman [13], and Grossman et al. [14] as shown in S1 Fig in S1 Appendix. The main model that is currently being extended in various dimensions has been developed by Antràs & Helpman [13]. This model derives its characteristics from two different models developed by [12] and Melitz [15] which have been extended by Grossman *et al*. [14], Kukharskyy [16], Gorodnichenko *et al*. [17], Cohle [18–20] in different dimensions.

Models developed by Williamson [21, 22], Grossman & Helpman [7], and Grossman & Hart [23] have addressed the role of transaction costs, incomplete contracts, and asset specificity that guide the decision of firms in choosing to undertake activity in-house or opt for outsourcing. An outsourcing model that addresses the issues of variations in cross-sectional and cross-regional outsourcing behavior of firms has been incorporated by Grossman & Helpman [7]. The authors argued that vertically integrated firms face higher cost of production as compared to specialized firms but search cost for collaborative outsourcing partners is high. Moreover, firms with different productivity levels go for different ownership structures. Higher productivity firms engage in foreign direct investment, medium productivity firms opt for foreign outsourcing while lower productivity firms operate domestically only. Standard trade model with imperfect competition and product differentiation characteristics have been incorporated in the outsourcing model by Antras [12] using the features from the [7, 21–24]. Whereas, firm heterogeneity was introduced in the dynamic industry model by Melitz [15], a commonly used model in the literature [13, 25]. Antràs & Helpman [13] developed a model of sourcing taking firm heterogeneity, product differentiation from [12, 15] in the North-South model of international trade in which final goods are developed in the North. This model serves as a basic model which has been extended by various researchers such as [14, 16–20]. Decision coordination between multinational firms and intermediate goods suppliers residing mainly in developing economies among culturally dissimilar countries where contracts are incomplete. The concept of rational contracts has been introduced by Kukharskyy [16] in the context of repeated games, which is a central part of foreign outsourcing collaboration. This research gap in the outsourcing model of Antràs & Helpman [13] has been bridged by Gorodnichenko *et al*. [17]. Whereas, R&D & innovation, intellectual property rights, and offshoring of innovation to south have been incorporated by the models reported in references [18–20]. The above theoretical literature indicates that most of the work on foreign outsourcing has been carried out from a developed economy perspective and no theoretical model exists from the developing economies perspective that directly analyzes the role of these firms to the best of our knowledge. In this regard, Hansen *et al*. [5] argued several theoretical domains indirectly shed light on foreign outsourcing from a developing economy firm’s perspective, they are usually approaching the issue from different perspectives such as a country and industry level perspective, and rarely explicitly apply a firm-level perspective. Moreover, they further emphasized that these models are inclined to view foreign outsourcing collaborations as functions of multinational corporation’s strategies, rather than strategies of developing economy firms. This implies that there is a need to have a model that analyzes the characteristics of firms from a developing economy firm’s perspective as no such models have been developed. Moreover, thousands of firms are evolving in developing countries because of outsourcing collaborations, creating jobs and export income, and contributing to the upgrading of skills and technology. Thus, a theoretical model focusing on foreign outsourcing collaboration from developing economy firms’ perspective is vital in determining their role in direct and indirect exports, attracting FDI through partial or concurrent foreign outsourcing collaborations.

## 3. Theoretical framework/model

A theoretical model is needed to understand the impact of firm characteristics, such as total factor productivity (TFP), knowledge spillovers, agglomeration economies, production, and social networks on foreign outsourcing collaborations from DEFs perspective. The literature indicates various economic models that have been developed to understand the nature of foreign outsourcing. However, such models have been developed from the perspective of developed economies. The most prominent work has been carried out by Grossman & Helpman [7], Antras [12], Antràs & Helpman [13], and Grossman *et al*. [14]. These models have been developed mainly to reflect the perspective of developed economies while developing economy perspectives have been incorporated marginally. Thus, we are compelled to develop a model that represents a DEFs perspective on foreign outsourcing collaborations.

Following Antràs & Helpman [13], the model assumes that the world consists of two economies which are the developed North and developing South. Consumers have identical preferences worldwide. Adapting the utility function of a representative consumer from Antràs & Helpman [13], which can be presented as

$$\begin{array}{cc} U=z_{0}+\frac{1}{\upsilon }\sum_{j=1}^{J}Z_{j}^{\upsilon } & 0<\upsilon <1 \end{array}$$ (1)

where, ***z***_**0**_ is the consumption of homogenous goods, ***Z***_***j***_ is an index of aggregate consumption of differentiated goods produced by sector ***j*** while ***υ*** is a parameter. The index of aggregate consumption is represented as,

$$\begin{array}{cc} Z_{j}={\left[\int z_{j}{\left(i\right)}^{\sigma }di\right]}^{\frac{1}{\sigma }} & 0<\sigma <1 \end{array}$$ (2)

where, ***z***_***j***_**(*i*)** is the consumption of different varieties. $\frac{1}{1-\sigma }$ is the elasticity of substitution between any two varieties while it is assumed that ***σ* > *υ***. The inverse demand function is

$$p_{j}\left(i\right)=\;Z_{j}^{\upsilon -\sigma }\;z_{j}{\left(i\right)}^{\sigma -1}$$ (3)

The assumptions of the model are as under:

1. Perfect supply of labor in both countries.
2. The wages in the North are assumed to be higher than wages in the South ***w***^***N***^ **> *w***^***S***^.
3. ***σ*** and ***υ*** are same in both countries.
4. Differences in technology and organizational costs between countries.
5. Only North knows how to produce final goods.

The production of any specific variety requires two variety-specific inputs.

1. $h_{f_{j}}\left(i\right)$ which is associated with headquarter services and can be produced only in the North.
2. ***m***_***j***_**(*i*)** which are manufactured components or intermediate inputs and can be produced both in the North and South.

Both variety-specific inputs can be produced with one unit of labor per unit of output in each country. Output final good for every variety produced in North is sector-specific Cobb-Douglas function as under

$$z_{j}\left(i\right)=\vartheta \;{\left[\frac{h_{f_{j}}\left(i\right)}{\rho_{j}}\right]}^{\rho_{j}}\;{\left[\frac{m_{j}\left(i\right)}{1-\rho_{j}}\right]}^{\rho_{j}}$$ (4)

where, ***ϑ*** represents productivity, ***ρ***_***j***_ shows sector-specific parameters. The larger the ***ρ***_***j***_ the more intensive sector is in headquarter services.

The model assumes two types of agents that supply inputs for the production of intermediate and final goods.

1. Final goods producers **(*H*)** residing in a foreign country who supply headquarter services ${(h}_{f_{j}})$ in the production of final goods in the North as well as in the production of intermediate goods in the South.
2. Operators of manufacturing plants **(*M*)** who supply headquarter services in the domestic economy **(*m***_***j***_**)** which are foreign outsourcing collaboration activities through which domestic firms supply intermediate goods to foreign outsourcing collaborating firms abroad.

As the model focuses on foreign outsourcing collaboration from the perspective of DEFs so the Cobb-Douglas production function of operators of manufacturing plants that produce and supply ***m***_***j***_ through operations in the developing economy South which is an output of firms in South (***Y***_***M***_**)** and is used as an input by firms in the North **(*m***_***j***_**)** and can be represented as

$$Y_{M}=\varphi \;N_{s}^{\alpha }\;h_{f}^{\beta }\;h_{p}{\left(F_{O_{i}}\right)}^{\gamma }$$ (5)

where, ***Y***_***M***_ is the output of operators of manufacturing plants which is used as an input ***m***_***j***_ for final good production in the North, ***φ*** is the productivity of firms, ***N***_***s***_ is the skilled labor supply or used by manufacturing plants in the production of intermediate goods, ***h***_***f***_ is input received from final goods producers. The vector of foreign headquarters services input ***h***_***f***_ received from final goods producers including design specifications, quality requirements, etc of intermediate goods outsourced. $h_{p}\left(F_{O_{i}}\right)$ is inputs other than labor including foreign outsourcing collaborations activities. The model assumes constant returns to scale (CRS).

The headquarter services function supplied by the domestic economy reflecting the vector of other inputs supplied by operators of manufacturing plants and vector of foreign outsourcing collaborating activities is given as,

$$h_{p}\left(F_{O_{i}}\right)=\alpha_{0}+\;\sum_{i=1}^{n}\beta_{i}F_{O_{i}}+\sum_{j=1}^{n}\beta_{j}F_{O_{j}}$$ (6)

The general form of production function is

$$Y_{M}=\varphi \left(N_{s},\;h_{f},\;h_{p}\left(F_{O_{i}}\right):\;Z_{p},\;E,\;S,\;A,\;X\right)$$ (7)

where, ***Z***_***p***_, ***E*, *S*, *A***, and ***X*** are the exogenous variables which are firm characteristics, efficiency, knowledge spillover composite variables, agglomeration economies composite variables, and direct exports respectively.

The cost constraint for the optimization problem is

$$C=wN_{s}+p_{hf}h_{f}+p_{hp}h_{p}\left(F_{O_{i}}\right)$$ (8)

where, ***w*** is the wages of the labor, ***p***_***hf***_ is the price of input supplied by headquarter services by the firm residing in the North, ***p***_***hp***_ is the price of composite input used by operators of manufacturing plants in the South.

Now the problem is to maximize revenue represented by

$$\text{max}\;R\;p_{M}\varphi \;N_{s}^{\alpha }\;h_{f}^{\beta }\;h_{p}{\left(F_{O_{i}}\right)}^{\gamma }$$ (9)

Subject to a cost constraint

$$C\;=wN_{s}+\;p_{hf}h_{f}+p_{hp}h_{p}\left(F_{O_{i}}\right)$$ (10)

The Lagrange for this optimization problem is

$$L=\;p_{M}\varphi \;N_{s}^{\alpha }\;h_{f}^{\beta }\;h_{p}{\left(F_{O_{i}}\right)}^{\gamma }+\;\lambda \;\left(\text{C}-wN_{s}-p_{hf}h_{f}-p_{hp}h_{p}\left(F_{O_{i}}\right)\right)$$ (11)

Using the Cobb–Douglas production function with elasticity coefficients, the first-order conditions are

$$w=\;\frac{\alpha p_{M}Y_{M}}{\lambda N_{s}}$$ (12)

$$p_{hf}=\;\frac{\beta p_{M}Y_{M}}{\lambda h_{f}}$$ (13)

$$p_{hp}=\frac{\gamma p_{M}Y_{M}}{\lambda h_{p}\left(F_{O_{i}}\right)}$$ (14)

$$C=wN_{s}+\;p_{hf}h_{f}+p_{hp}h_{p}\left(F_{O_{i}}\right)$$ (15)

Dividing (12) by (13) we get,

$$N_{s}=\frac{\alpha \;p_{hf}h_{f}}{\beta w}$$ (16)

$$h_{f}=\frac{\beta \;w\;N_{s}}{\alpha \;\;p_{hf}}$$ (17)

Dividing (12) by (14) we get,

$$N_{s}=\frac{\alpha \;p_{hp}\;h_{p}\left(F_{O_{i}}\right)}{\gamma \;w}$$ (18)

$$h_{p}\left(F_{O_{i}}\right)=\frac{\;\gamma \;w\;N_{s}}{\alpha \;p_{hp}}$$ (19)

Substitute (16) in cost constraint (15)

$$p_{hf}h_{f}=\;\left(\frac{\beta }{\alpha +\beta }\right)\left[C-p_{hp}h_{p}\left(F_{O_{i}}\right)\right]$$ (20)

which yields demand for headquarter services received from final goods producers as

$$h_{f}=\left(\frac{\beta }{\alpha +\beta }\right)\left[\frac{C-p_{hp}h_{p}\left(F_{O_{i}}\right)}{p_{hf}}\right]$$ (21)

In general form, it can be written as

$$h_{f}=f\left(w,\;\;p_{hf},p_{hp},N_{s},h_{p}(F_{O_{i}}),\;C:\;Z_{p},\;E,\;S,\;A,\;X\right)$$ (22)

The corresponding inverse demand function is

$$p_{hf}=\;\left(\frac{\beta }{\alpha +\beta }\right)\left[\frac{C-p_{hp}h_{p}\left(F_{O_{i}}\right)}{h_{f}}\right]$$ (23)

The general form can be written as

$$p_{hf}=\;f\left(w,\;p_{hp},N_{s},h_{f},h_{p}(F_{O_{i}}),\;C:\;Z_{p},\;E,\;S,\;A,\;X\right)$$ (24)

Substitute (17) in cost constraint (15)

$$N_{s}=\;\left(\frac{\alpha }{\alpha +\beta }\right)\left[\frac{C-p_{hp}h_{p}\left(F_{O_{i}}\right)}{w}\right]$$ (25)

$$N_{s}=\;f\left(w,\;\;p_{hf},p_{hp},h_{f},h_{p}(F_{O_{i}}),\;C:\;Z_{p},\;E,\;S,\;A,\;X\right)$$ (26)

$$w=\;\left(\frac{\alpha }{\alpha +\beta }\right)\left[\frac{C-p_{hp}h_{p}\left(F_{O_{i}}\right)}{N_{s}}\right]$$ (27)

$$w=\;f\left(p_{hf},p_{hp},N_{s},h_{f},h_{p}(F_{O_{i}}),\;C:\;Z_{p},\;E,\;S,\;A,\;X\right)$$ (28)

Now substitute (18) in cost constraint (15)

$$h_{p}\left(F_{O_{i}}\right)=\;\left(\frac{\gamma }{\alpha +\gamma }\right)\left[\frac{C-p_{hf}h_{f}}{p_{hp}}\right]$$ (29)

The general form can be written as

$$h_{p}(F_{O_{i}})=\;f\left(w,\;\;p_{hf},p_{hp},N_{s},h_{f},\;C:\;Z_{p},\;E,\;S,\;A,\;X\right)$$ (30)

The corresponding inverse demand function is

$$p_{hp}=\;\left(\frac{\gamma }{\alpha +\gamma }\right)\left[\frac{C-p_{hf}h_{f}}{h_{p}\left(F_{O_{i}}\right)}\right]$$ (31)

The general form can be written as

$$p_{hp}=\;f\left(w,\;\;p_{hf},h_{f},\;N_{s},h_{p}\left(F_{O_{i}}\right),\;C:\;Z_{p},\;E,\;S,\;A,\;X\right)$$ (32)

## 4. Comparative Statics Analysis (CSA)

This section provides the results of comparative static analysis. This analysis entails solving the necessary conditions given in Eqs (21) and (29) by using the methodology provided by Gale & Nikaido [26] which yields a Jacobian matrix in the form of the system of equations and the final equations are follows.

$$X=[\begin{array}{cccccc} \frac{\psi N_{s}}{p_{hf}} & \frac{\psi (p_{hp}h_{p}(F_{O_{i}})-C)}{p_{hf^{2}}} & -\frac{\psi h_{p}\left(F_{O_{i}}\right)}{p_{hf}} & 0 & -\frac{\psi p_{hp}}{p_{hf}} & \frac{\psi }{p_{hf}}\\ \frac{\varrho N_{s}}{p_{hp}} & -\frac{\varrho h_{f}}{p_{hp}} & \frac{\varrho \left(p_{hf}h_{f}-C\right)}{p_{hp^{2}}} & -\frac{\varrho p_{hf}}{p_{hp}} & 0 & \frac{\varrho }{p_{hp}} \end{array}]$$ (33)

where $\psi =\frac{\beta }{\alpha +\beta }$ and $\varrho =\frac{\gamma }{\alpha +\gamma }$

$$Y=[\begin{array}{cc} \frac{\partial F_{1}}{\partial h_{f}} & \frac{\partial F_{1}}{\partial h_{p}(F_{O_{i}})}\\ \frac{\partial F_{2}}{\partial h_{f}} & \frac{\partial F_{2}}{\partial h_{p}(F_{O_{i}})} \end{array}]=[\begin{array}{cc} \frac{\beta (\beta -1)p_{M}Y_{M}}{{h_{f}}^{2}} & \frac{\beta \gamma p_{M}Y_{M}}{h_{f}h_{p}(F_{O_{i}})}\\ \frac{\beta \gamma p_{M}Y_{M}}{h_{f}h_{p}(F_{O_{i}})} & \frac{\gamma (\gamma -1)p_{M}Y_{M}}{h_{p}{\left(F_{O_{i}}\right)}^{2}} \end{array}]$$ (34)

$$\left|Y\right|=\;\left(-\beta \gamma \left(\beta +\gamma -1\right)\right)\;\left[\frac{{p_{M}}^{2}{Y_{M}}^{2}}{{h_{f}}^{2}h_{p}{\left(F_{O_{i}}\right)}^{2}}\right]\;>0$$ (35)

which **(|*Y*| > 0)** holds under the condition of CRS whereas it can be negative under increasing returns to scale.

### Comparative analysis of domestic inputs and foreign outsourcing collaborations decision in production of intermediate goods

**Proposition 01**: All other things being unchanged, an increase in the wages of skilled labor would decrease the demand for local inputs & domestic headquarter services by the operators of manufacturing plants and reduce foreign outsourcing collaborations.


$$\frac{\partial h_{p}(F_{O_{i}})}{\partial w}=\;\frac{\beta N_{s}p_{M}Y_{M}\left[\left(\beta -1\right)\varrho p_{hf}h_{p}(F_{O_{i}})-\gamma \psi p_{hp}h_{f}\right]/p_{hf}p_{hp}{h_{f}}^{2}h_{p}(F_{O_{i}})}{\left|Y\right|}<0$$ (36)

**Fig 1:**
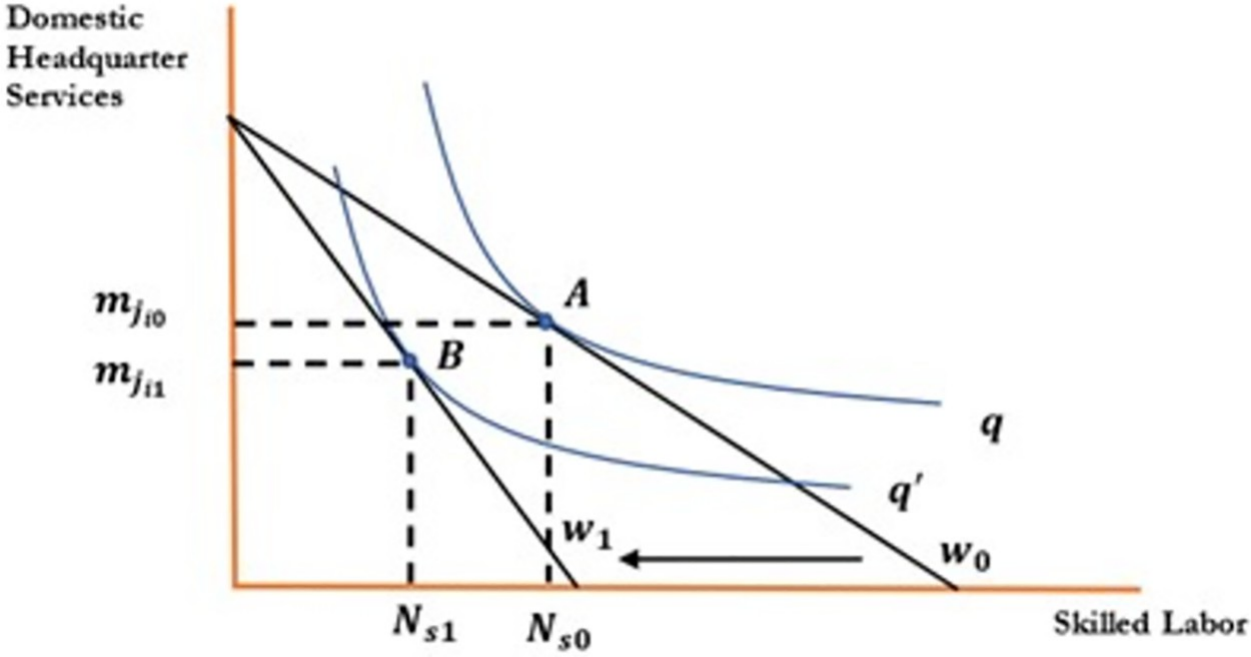
Relationship between foreign outsourcing collaboration and skilled employees’ wages.

**Fig 2:**
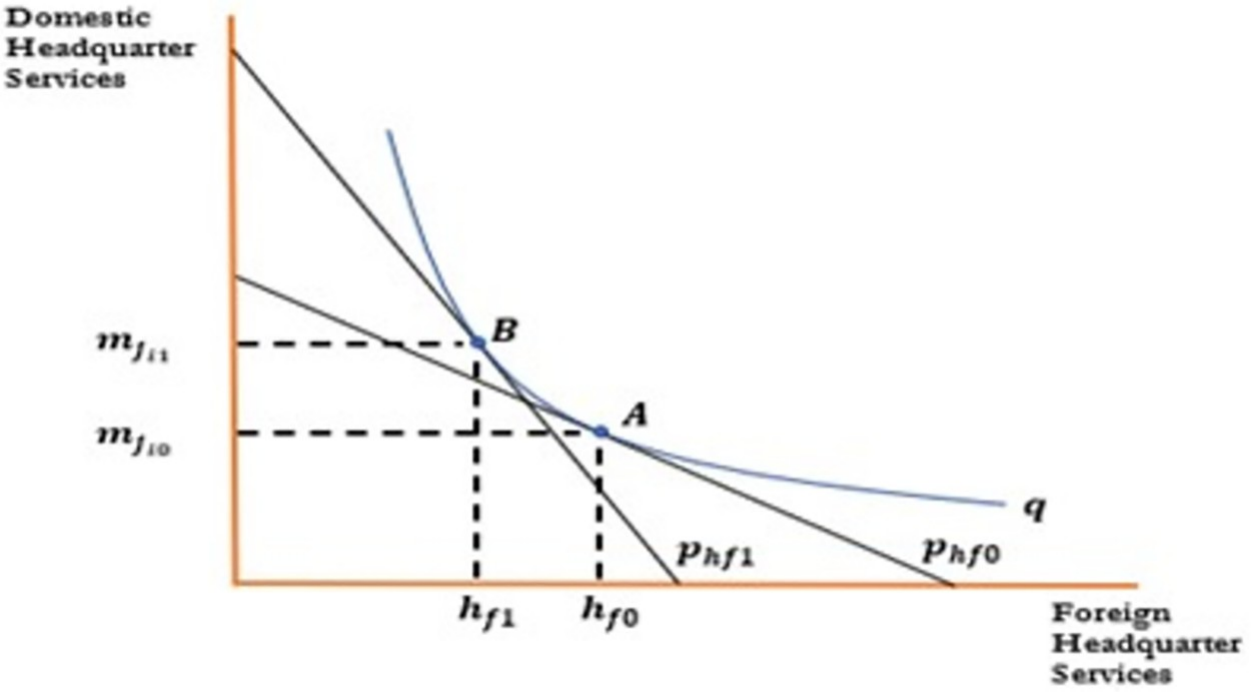
Relationship between foreign outsourcing collaboration and foreign headquarter services.

**Fig 3:**
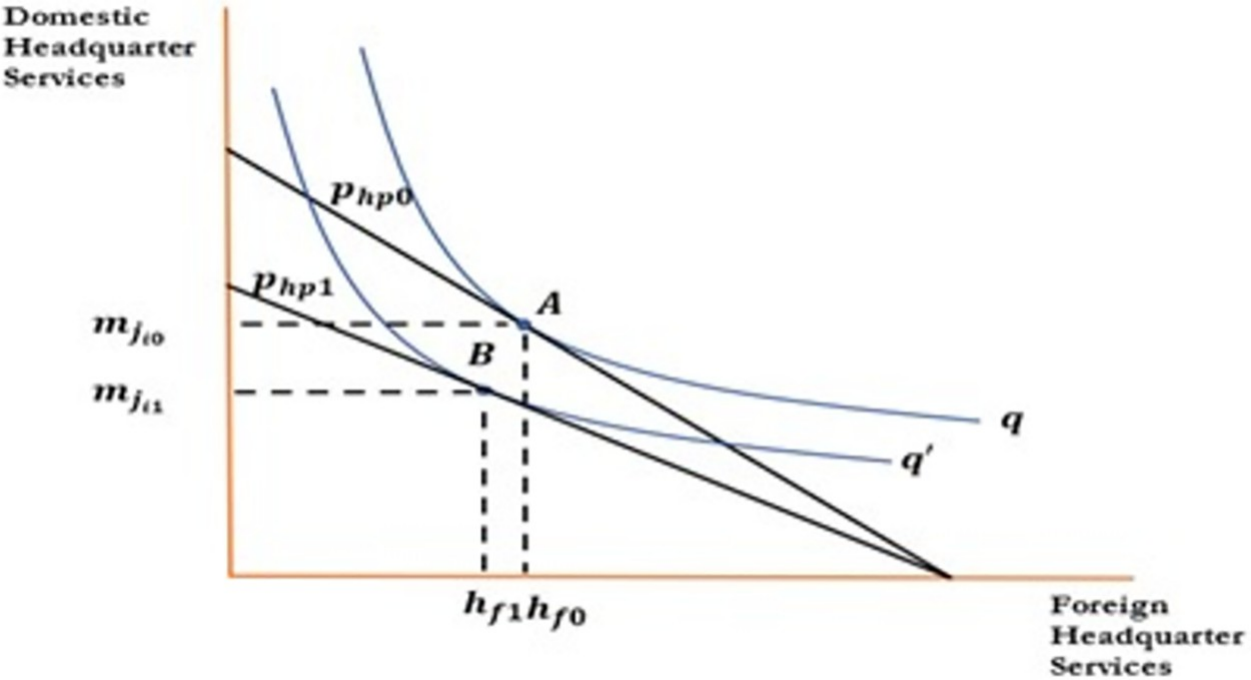
Relationship between foreign outsourcing collaboration and domestic headquarter services.

**Fig 4:**
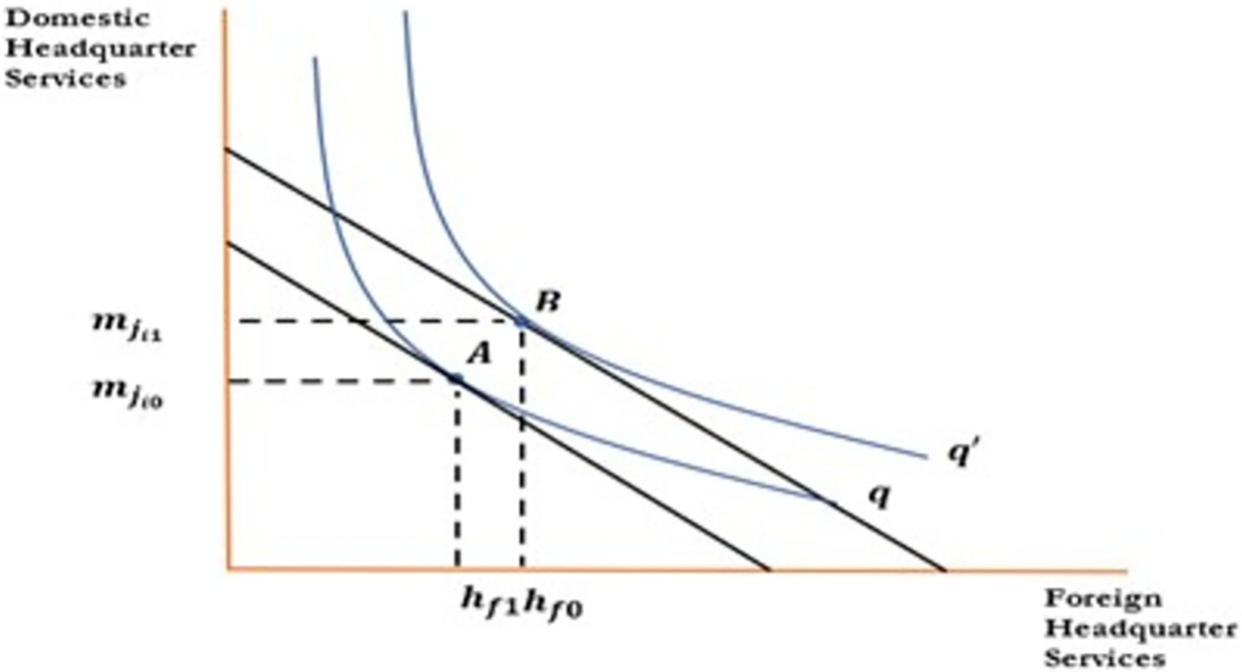
Relationship between foreign outsourcing collaboration and demand for foreign headquarter services.

Fig 1 indicate the effect of change in wages of skilled workers on foreign outsourcing collaboration. To draw the relationship between the aforementioned variables, the horizontal axis represents skilled labore; the vertical axis represents domestic headquarter services. An increase in wages decreases demand for domestic inputs, which rotate the isocost line inward. An increase in wages decreases demand for domestic inputs decreasing skilled labor from *N*_*s*0_ to *N*_*s*1_ and domestic headquarter services from $m_{j_{i0}}$ to $m_{j_{i1}}$. Firms will be reluctant to collaborate in foreign outsourcing as an increase in wages increases their cost of production and decreases profits that move optimum point from A to B. An increase in wages reduces the wage differential between the North and South, which discourages firms to outsource activities to the firms in the South. Hence an increase in wages negatively affects the foreign outsourcing decision of the firms in the South as shown in Fig 1 and Eq (36). In a similar fashion, Figs 2 to 4 have been drawn and interpreted accordingly to postulate the effects of corresponding variable on the foreign outsourcing collaboration. Figs 3 and 4.

**Proposition 02**: All other things being unchanged, an increase in the price of foreign headquarter services or foreign input would increase the demand for domestic inputs and **increase** foreign outsourcing collaborations.


$$\frac{\partial h_{p}(F_{O_{i}})}{\partial p_{hf}}=\frac{\beta p_{M}Y_{M}\left[-\left\{\left(\beta -1\right)\varrho {p_{hf}}^{2}h_{p}(F_{O_{i}})\right\}-\left\{\psi \left(p_{hp}h_{p}\left(F_{O_{i}}\right)-C\right)\right\}\left(\gamma p_{hp}\right)\right]/h_{f}\;{p_{hf}}^{2}h_{p}(F_{O_{i}})}{\left|Y\right|}>0$$ (37)

**Proposition 03**: All other things being unchanged, an increase in the price of domestic inputs and headquarter services would decrease the demand for domestic inputs and **decreases** foreign outsourcing collaborations.


$$\frac{\partial h_{p}(F_{O_{i}})}{\partial p_{hp}}=\;\frac{\gamma p_{M}Y_{M}\left[\psi \left(\gamma -1\right){p_{hp}}^{2}h_{f}-\beta \varrho p_{hf}\left(p_{hf}h_{f}-C\right)\right]/p_{hf}h_{f}{p_{hp}}^{2}h_{p}(F_{O_{i}})}{\left|Y\right|}<0$$ (38)

**Proposition 04**: All other things being unchanged, an increase in demand for foreign headquarter services and inputs would increase the demand for domestic inputs and increases foreign outsourcing collaborations.


$$\frac{\partial h_{p}(F_{O_{i}})}{\partial h_{f}}=\;\frac{\left[-\beta \left(\beta -1\right)\varrho p_{hf}p_{M}Y_{M}\right]/p_{hp}{h_{f}}^{2}}{\left|Y\right|}>0$$ (39)

An increase in demand for foreign headquarter services and inputs has a direct impact on the quality of products being produced. The improvement in the quality of goods would be translated into increased demand for products being produced and delivered. Thus, when firms increase the demand for domestic inputs they would also increase foreign outsourcing collaborations.

**Proposition 05**: All other things being unchanged, an increase in the cost of production would decrease the demand for domestic inputs and decrease foreign outsourcing collaborations.


$$\frac{\partial h_{p}(F_{O_{i}})}{\partial C}=\;\frac{\beta p_{M}Y_{M}\left[\varrho \left(\beta -1\right)p_{hf}h_{p}(F_{O_{i}})-\gamma \psi p_{hp}h_{f}\right]/p_{hf}{h_{f}}^{2}p_{hp}h_{p}(F_{O_{i}})}{\left|Y\right|}<0$$ (40)

An increase in the cost of production negatively affects the level of a firm’s production as it the decreases firm’s revenue and profit level. The decrease in the firm’s level of production would decrease the demand for domestic inputs and decreases foreign outsourcing collaborations.

## 5. Material, methods, and model

Ideally, one might use secondary data on the aforementioned factors to test the theoretical relationship of the firm model created in Section 3 practically. However, a significant issue, particularly in the case of developing countries, is the accessibility of the data at the national, regional, and local levels. For instance, data on firms that are involved in foreign outsourcing collaboration is not available in any firm-level survey conducted in the case of Pakistan and other developing economies. Data regarding the involvement of firms in direct, indirect exports, firms that serve only domestic markets, foreign and domestic headquarter services can only be collected through the firm survey. Through a firm survey, we have gathered data on the variable that is essential to our theoretical model to undertake an econometric analysis. We have increased confidence that the exogenous variable in the model can account for most of the foreign outsourcing collaboration.


$$h_{p}(F_{O_{i}})=\;f\left(w,\;\;p_{hf},p_{hp},N_{s},h_{f},\;C:\;Z_{p},\;E,\;S,\;A,\;X\right)$$ (41)

The empirical model is defined as follows: the foreign outsourcing collaboration is a function of wages of skilled labor, price of foreign headquarter services or foreign input, price of domestic inputs and headquarter services, skilled labor supply or skilled labor used by manufacturing plants in the production of intermediate goods, vector of foreign headquarters services input ***h***_***f***_ received from final goods producers include design specifications, quality requirements, etc of intermediate goods outsourced and exogenous variables which are firm characteristics, efficiency, knowledge spillover composite variables, agglomeration economies composite variables, and direct exports.

From the theoretical model, we know that wages of skilled labor, price of domestic inputs or domestic headquarter services, and cost of production are negatively related to foreign outsourcing collaboration, therefore it is expected that from the empirical analysis, we get a similar relationship. Similarly, the price of foreign input or foreign headquarter services, and demand for foreign headquarter services & inputs positively affect foreign outsourcing collaboration, in the same way, we are hoping to get the desired results empirically.

The firm survey was conducted in the peripheries of Faisalabad which is one of Pakistan’s largest cities of Pakistan known as the “Manchester of Pakistan” due to its importance in the country’s textile industry. The textile industry is the largest industry in Faisalabad, contributing significantly to the city’s economy and job creation. In Faisalabad, numerous textile mills produce yarn, fabric, and apparel as well as spinning and weaving mills, dyeing, printing mills, and finishing mills.

The background knowledge of firm characteristics and geographic agglomeration of firms was gained when selecting the study area. This consideration enabled us to identify the firm clusters in Faisalabad which are Small Industrial Estate, Millat Industrial Estate, Khurianwala, Sargodha Road, Sheikhupura Road, Canal Road, and Jaranwala Road Faisalabad. Then, within this area of study, new information through the field survey using a structured questionnaire was collected, which was structured according to **4** main topics among others: 1. Firm Characteristics; 2. Foreign Outsourcing Collaborations; 3. Innovation; 4. Firm’s Networking, Affiliation, Agglomeration, and Economic Policy.

Information from 217 firms and units was collected and analyzed to access the relation of firm characteristics with the likelihood of engaging in foreign outsourcing collaboration. Demographic variables for the firm considered were firm size, firm age, and educational level of the owner if the owner is managing the organization. Whereas firm operation and income generation activities were identified through firm involvement in foreign and domestic business activity, i.e., direct exports and indirect exports through foreign outsourcing collaboration were considered for foreign business activity, and domestic outsourcing collaboration and local business only were considered for domestic business activity.

Before conducting the full firm survey, a pilot study was conducted in Faisalabad. A full field survey was conducted from July 17 to August 31, 2023, employing standardized methods [27] to minimize interviewing errors. The ethics committee of the School of Social Sciences and Humanities (S3H), National University of Sciences and Technology (NUST), Islamabad, Pakistan, provided a formal ethical approval prior to the conduct of the survey vide letter number 0988/Ethic/01/S3H/131/ECO. The committee recommended that there is no need to obtain informed consent as the survey includes textile and apparel firms. Basic data based on the firm and proprietor name, and National Tax Number (NTN) and address were obtained from All Pakistan Bedsheets & Upholstery Manufacturer’s Association (APBUMA) which provided extraordinary support during the field survey. Additionally, the firms having membership with other associations were also obtained from their respective online sources such as All Pakistan Textile Mills Association (APTMA) and Faisalabad Chamber of Commerce and Industry (FCCI). Nonetheless, despite several visits by the authors to FCCI, and other associations, we did not receive any assistance for field visits. The apparent lack of collaboration from these organizations has significantly affected survey responses.

The accumulated share of both direct and indirect exporting firms is 61.75% as compared to firms that are not involved in either type of export as indicated in S1 Table in S2 Appendix. Most of the firms that are engaged in both direct and indirect exports through foreign outsourcing collaboration are large (35.18%) followed by medium size 16.59% and small size 11.98%.

S2 Table in S2 Appendix shows the proportion of exporting and non-exporting firms along with the firm’s legal ownership status and firm size. About 61.75% of firms are engaged in exports of textile and apparel goods out of which 23.50, 16.59, 14.75, and 6.91 percent are private limited liability companies, sole proprietorship, partnership, and public limited liability companies respectively. It can be seen in S2 Table in S2 Appendix that the majority of the firms that are engaged in exporting activity are large and their legal ownership status is private limited liability company (i.e., 21.2%) and public limited liability company (i.e., 6.91%). The results further reveal that the majority of firms that are not involved in exports are sole proprietary firms and are small which constitutes 18.89% of total sole proprietor firms i.e., 26.27%.

S3, S4 Tables in S2 Appendix indicate the share of firms that engaged in direct and indirect exporting activity through foreign outsourcing collaboration and are bifurcated with respect to legal ownership status. S3 Table in S2 Appendix indicates that 33.64% of total firms are involved in direct exports of textile and apparel goods while 66.36% are not involved in direct exports while S4 Table in S2 Appendix shows that 35.02% of the total are engaged in indirect exports. Whereas, S1 Table in S2 Appendix illustrates that firms that are engaged in both types of exporting activities are 61.75%. S3 Table in S2 Appendix further indicates that most of the firms that are engaged in direct exporting activities are sole proprietors while the private limited liability company that are mostly involved in indirect exporting activities through foreign outsourcing collaborations.

To estimate the Eq (41) variables including price of foreign headquarter services/inputs were excluded as the firm does not pay any sum of money in return for receiving various inputs from the final good producer directly, however, they must pay overhead costs for meeting the specifications and requirements provided. Thus, such overhead costs may be used as a proxy for the price of foreign headquarter services or foreign input. The unavailability of data on price of foreign headquarter services/input compelled us to drop these variables as firms were reluctant to share such information. Model 1 explains the main determinants of foreign outsourcing collaboration. Distribution/transportation and marketing activities have been incorporated in Model 2 and 3 respectively, as exogenous factors (S9 Table in S2 Appendix). It turned out that marketing helps textiles and apparel firms to expand their scope of products, to comprehend foreign outsourcing collaborators dynamics, and grasp customer preferences. The marketing activities have been included in Model 2 due to their significant impact on foreign outsourcing collaboration. Effective marketing facilitates the developing economy’s firms to increase indirect exports through market penetration, and access to new markets. Model 3 includes transportation/distribution activities, which reduce logistics costs, enhance the efficiency of developing economy firms, and enable them to gain competitive advantage. The surveyed firms identified their top 8 export destinations as: USA, UK, Italy, Spain, Germany, France, Australia, and Canada based on export destinations disclosed by firms as provided in S5 Table in S2 Appendix.

The optimal amount or threshold value beyond which any increase in the proportion of the cost of production will result in a decline in international outsourcing cooperation is reflected by the tipping point. This tipping point can be determined by assessing the non-linearity of the relationship between the dependent (foreign outsourcing collaborations) and independent variables (share of the cost of sales in total production), by the addition of the squared variable share of the cost of sales in total production, as shown in the model 1.

To find the estimated value of the tipping point for temperature, for instance, the estimated regression for model 1 was used as under:

$$h_{p}(F_{O_{i}})=\beta_{0}+\beta_{1}w_{lp}+\beta_{2}N_{s}+\beta_{3}h_{f}+\beta_{4}C_{s}+\beta_{5}C_{s}^{2}+\beta_{6}V+\beta_{7}h_{p}+\beta_{8}+\varepsilon$$ (42)

Once the above model is estimated using OLS, the tipping point is calculated using the following formula.


$$\frac{dh_{p}(F_{O_{i}})}{dC_{s}}=\;\beta_{4}+2\beta_{5}\left(C_{s}\right)=0$$ (43)

$$C_{s}=\;-\frac{\beta_{4}}{2*\beta_{5}}$$ (44)

The econometric valuation approach needs a dependent variable which is the firm’s indirect exports through foreign outsourcing collaboration. Firms generally export goods and services; however, standard enterprise surveys usually do not ask firms to report data on indirect exports through foreign outsourcing collaboration. This study used the proportion of total exports linked to indirect exports for measuring the involvement of firms in foreign outsourcing collaborations.

## 6. Results and discussion

A total of 240 firms have been surveyed and 217 have been included in empirical estimation after removing outliers from the data. The average firm is found to have 501.27 employees. The dependent variable is the proportion of total exports linked to indirect exports, that is, exports through foreign outsourcing collaboration. The dependent variables can take any value from 0 to 100% or 0 to 1 so the most appropriate estimation method available is Ordinary Least Square (OLS). The variables and their measurement are provided in S6 Table in S2 Appendix. The descriptive statistics are shown in S7 Table in S2 Appendix while the results of the OLS regression analysis are given in S9 Table in S2 Appendix. Prior to regression analysis, all three models have been tested for the presence of heteroscedasticity which are provided in S8 Table in S2 Appendix. The results confirm its presences which has been tackled by employing robust standard errors (SEs).

### Wages

The wage to labor-productivity ratio measures how much workers are paid in relation to how much they produce. It is calculated by dividing the wages to labor-productivity paid by textile and apparel firms during fiscal year 2022. Generally, wage to labor productivity ratio greater than 1 implies that workers are paid more than they produce, a wage to labor productivity ratio less than 1 implies that workers are paid less than they produce, while a wage to labor productivity ratio equal to 1 implies that workers are paid in accordance with their productivity.

The impact of wage to labor productivity ratio has a negative and significant impact on foreign outsourcing collaboration. The coefficient for wage to labor productivity indicates that for every 1% increase in the wage to labor productivity ratio, the percentage of indirect exports related to foreign outsourcing collaboration decreases by 0.000133 percent points. The negative impact of wage to labor productivity implies that higher the wage to labor productivity lowers the foreign outsourcing collaborations. This further implies that firms with higher wage to labor productivity ratio are paying higher wages to their employees, which in turn reduces demand for other domestic inputs as a result foreign outsourcing collaboration would decrease.

### Foreign headquarters services

Foreign headquarters services input ***h***_***f***_ received from final goods producers including design specifications, quality requirements, etc of intermediate goods outsourced. As hypothesized in the economic model, that increase in the price of foreign headquarter services or foreign input would increase the demand for domestic inputs and increases foreign outsourcing collaborations. The empirical results are consistent with the economic model that an increase in foreign headquarter services would positively and significantly affect foreign outsourcing collaboration. A 1-percentage point increase in foreign headquarters services would increase 0.224 percentage point in foreign outsourcing collaboration.

### Cost of production

The share of the cost of sales in total production refers to the proportion of expenditures incurred in the production of goods divided by total production. It represents the portion of the total cost of production that can be allocated to the production of goods in the textile and apparel industry. The empirical estimation also includes its nonlinear term in form of a square term to determine the inverted U-shaped relationship between the proportion of cost and foreign outsourcing collaborations. The inverted U-shaped is ascertained by the empirical estimation, which shows that the coefficient of the level term i.e., the share of the cost of sales in total production is positive and significant 5% level of significance while its square is negative and significant at 10% level of significance. Such a relationship between independent and dependent variables is characterized as an inverted U-shaped relationship. Thus, it is crucial to keep in mind that to completely comprehend the relationship, the coefficient’s value must be understood in conjunction with the squared term.

### Tipping point

While the regression results for Model 1 indicate that ***β***_**4**_ is 0.953 and ***β***_**5**_ is -0.663 so the tipping point is calculated as

$$C_{s}=\;-\frac{0.953}{2*\left(-0.663\right)}=0.719$$ (45)

It implies that the relationship between the share of cost and the percentage of foreign outsourcing collaboration reverses once it reaches a value of about 0.719. Prior to this turning point, a rise in the cost share is associated with a rise in the export share. Beyond the tipping point, however, continued rises in the cost share would lead to a decline in the foreign outsourcing collaboration share.

### Domestic inputs

Domestic inputs in developing economies are key factors that enable foreign firms to collaborate with developing economies’ domestic firms. The results indicate that the use of domestic inputs negatively affects foreign outsourcing collaboration; however, this impact is insignificant. This implies that firms that use domestic raw materials such as cotton play no role in attracting foreign outsourcing collaboration.

### Skilled labor

Foreign firms collaborate in terms of outsourcing with firms in developing economies due to the availability of skilled labor. In this context, the total number of skilled employees (having skill certification) is included in the model. The coefficient of skilled employees is positive and significant which implies that skilled employees are a significant determinant of foreign outsourcing collaborations.

### Economies of scope/ variety of goods

The total number or variety of goods produced by the firm is included as the control variable in the model. A firm that produces more variety can benefit from economies of scope. The results indicate that firms that produce more variety are engaged more in foreign outsourcing collaborations. This further implies that economies of scope are a significant determinant of foreign outsourcing collaborations.

### Distribution/transportation and marketing activities

In models 2 and 3, we have included distribution/transportation and marketing activities in achieving competitive advantage. Both variables are insignificant meaning that neither distribution/transportation activities nor marketing activities are important in attracting foreign outsourcing collaboration.

### Diagnostic testing

The models have a problem of heteroskedasticity which is corrected with the use of robust standards errors. We have also tested for multicollinearity using VIF and model specification using the Ramsey RESET test for omitted variables, the test results are shown in S8, S9 Tables in S2 Appendix. The results indicate that the model has no problem of multicollinearity as values of VIF for all the variables are well below 10 and the mean VIF is 1.52.

We have applied the Ramsey RESET test for powers of independent variables as our model is nonlinear. The null hypothesis for the test is model has no omitted variables while the alternative hypothesis is model has omitted variables. The probability of the F-test is 0.3952 which indicates that we cannot reject the null hypothesis of no omitted variables. Thus, the Ramsey RESET test for powers of independent variables indicates that the model is correctly specified.

## 7. Conclusion and policy implications

### Conclusion

This study has developed an economic model from the developing economy firms’ perspective to delineate the determinants of foreign outsourcing collaboration to address the problem of low exports and enhance the competitive advantage. In this context, firm-specific characteristics are analyzed concerning foreign outsourcing collaboration decisions made by firms. Empirical validation of the model has been carried out by using the primary data collected from textile and apparel firms operating in the city of Faisalabad, Pakistan. 217 firms have been surveyed.

The results indicate that the wage to labor-productivity ratio negatively affects foreign outsourcing collaboration thus suggesting that the firms having higher wage to labor productivity ratio are paying higher wages which lowers demand for domestic inputs other than labor as a result foreign outsourcing collaboration would decrease. The share of cost in total production makes an inverted U-shape relationship via foreign outsourcing collaboration. It thus indicates that at the initial stage, the firm’s cost of production increases with an increase in the level of foreign outsourcing collaborations. Whereas after the tipping point, which is at 0.719 as a share of the cost of sales, the firm’s cost starts decreasing with a further increase in foreign outsourcing collaborations. Furthermore, the demand for skilled labor, foreign headquarters services, and variety being produced by the firm (i.e., economies of scope) are positively associated with foreign outsourcing collaboration. It is worth noting that domestic inputs play no role in attracting foreign firms in outsourcing collaboration which may be due to the low quality of domestic inputs available such as the short length of the cotton lint. Similarly, the weak distribution/transportation and marketing networks make outsourcing collaborations between foreign and local firms fragile.

### Policy implications

From the aforementioned conclusion, the following implications are dawn for policy:

i Sharply increase labor productivity by developing high-end skills and by improving labor proficiency to work according to the fast-changing requirements of foreign outsourcing collaborators.
ii Firms need to focus on achieving the economies-of-scope using the same raw materials or manufacturing plants and facilities. For example, firms producing apparel can use discarded materials from the main product production to make other small products or converting into yarn rather than wasting them.
iii Use improved technology and know-how to produce high quality domestic inputs to ensure the export of cost-effective and better-quality final products to foreign collaborators.
iv Firms need to focus on strengthening their distribution/transportation and marketing networks to ensure timely cost-effective supplies.

Firms require using improved domestic inputs that satisfy international standards to become more cost effective and competitive. The government ought to promote the use of domestic inputs that enhance firms’ productivity and improve wags to labor productivity conditions within the textile and apparel industry and economy at large. Such initiatives can be brought about by focusing more on domestic headquarter services instead of foreign headquarter services. More foreign headquarter servicers should be attracted in areas that are new to the developing economy enterprises such as innovation, and marketing activities. This results in economies of scope due to the involvement of enterprises in a broad array of products. Furthermore, the government should bridge the geographical barriers by conducting a combo of virtual and on-site meetings to understand the culture and business practices of outsourcing partners. The government needs to also facilitate firms to gain specific marketing knowledge through participation in trade shows and exhibitions at the international level as well as organize such trade shows domestically.

## Supporting information

S1 Appendix Literature review. (DOCX)

S2 Appendix Summary statistics and empirical results. (DOCX)

S1 Questionnaire (PDF)
