# Supplementary figures and images for: Foreign outsourcing collaboration within a developing economy’s perspective: A case of the Pakistani textile industry
Source: PLoS One. 2024 Apr 16;19(4):e0299454. doi: 10.1371/journal.pone.0299454 (PMC11020694; doi:10.1371/journal.pone.0299454)

**S1 Appendix:**

Fig 1: Literature Review


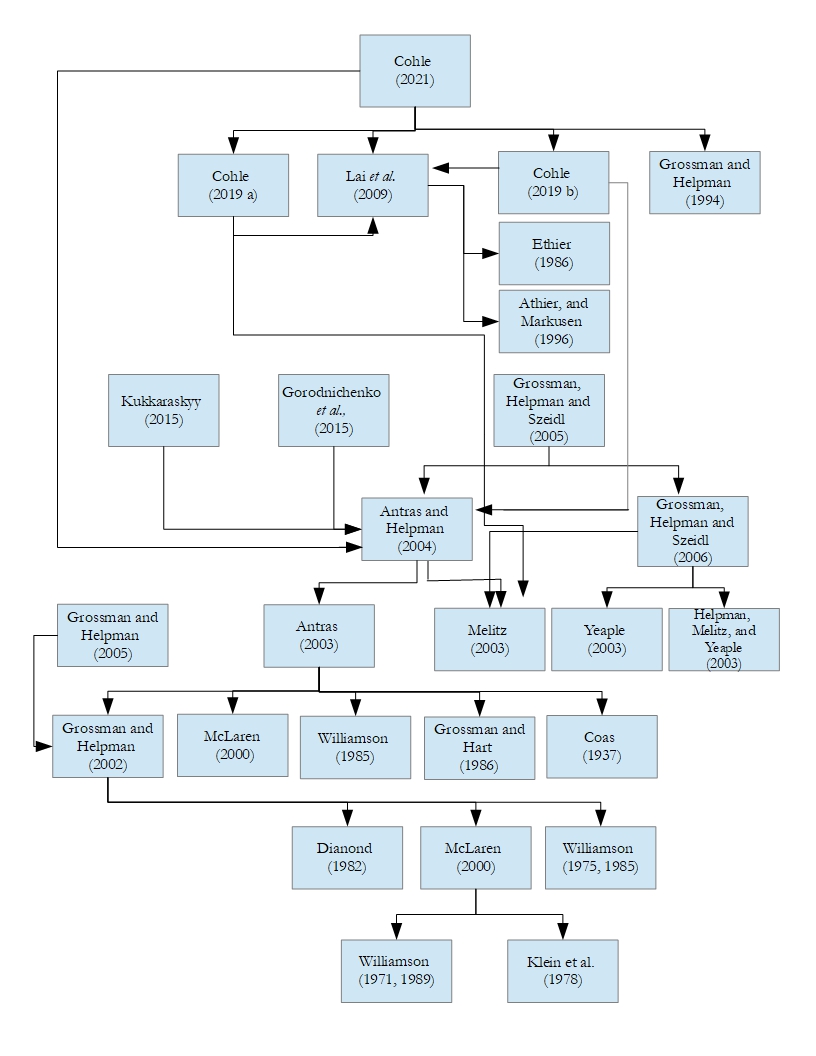


**Source:** Authors’ Own Conception

Supplement: S1 Appendix — (DOCX) [file pone.0299454.s001.docx]
