# Supplementary material for: Foreign outsourcing collaboration within a developing economy’s perspective: A case of the Pakistani textile industry
Source: PLoS One. 2024 Apr 16;19(4):e0299454. doi: 10.1371/journal.pone.0299454 (PMC11020694; doi:10.1371/journal.pone.0299454)
Supplement: S2 Appendix — (DOCX) [file pone.0299454.s002.docx]

**S2 Appendix:**

S2 Table 1: Share of Direct and Indirect Exporting Firms and Firm Size

| **Firm Size** | **Yes** | **No** | **Grand Total** |
| --- | --- | --- | --- |
| Small | 11.98% | 24.42% | 36.41% |
| Medium | 16.59% | 9.68% | 26.27% |
| Large | 33.18% | 4.15% | 37.33% |
| **Grand Total** | **61.75%** | **38.25%** | **100.00%** |

S2 Table 2: Share of Total Exports in Total Production, Ownership Status, and Firm Size

| **Ownership Status and Firm Size** | **Yes** | **No** | **Grand Total** |
| --- | --- | --- | --- |
| **Sole Proprietorship** | **16.59%** | **26.27%** | **42.86%** |
| Small | 6.91% | 18.89% | 25.81% |
| Medium | 6.45% | 5.53% | 11.98% |
| Large | 3.23% | 1.84% | 5.07% |
| **Partnership** | **14.75%** | **8.76%** | **23.50%** |
| Small | 4.61% | 5.53% | 10.14% |
| Medium | 8.29% | 2.30% | 10.60% |
| Large | 1.84% | 0.92% | 2.76% |
| **Private Ltd Liability Company** | **23.50%** | **1.38%** | **24.88%** |
| Small | 0.46% | 0.00% | 0.46% |
| Medium | 1.84% | 0.46% | 2.30% |
| Large | 21.20% | 0.92% | 22.12% |
| **Public Ltd Liability Company** | **6.91%** | **1.84%** | **8.76%** |
| Medium | 0.00% | 1.38% | 1.38% |
| Large | 6.91% | 0.46% | 7.37% |
| **Grand Total** | **61.75%** | **38.25%** | **100.00%** |

S2 Table 3: Share of Direct Exporting Firms and Ownership Status

| **Ownership Status of Direct Exporting Firm** | **Yes** | **No** | **Grand Total** |
| --- | --- | --- | --- |
| Sole Proprietorship | 11.98% | 30.88% | 42.86% |
| Partnership | 9.68% | 13.82% | 23.50% |
| Private Ltd Liability Company | 7.37% | 17.51% | 24.88% |
| Public Ltd Liability Company | 4.61% | 4.15% | 8.76% |
| Grand Total | 33.64% | 66.36% | 100.00% |

S2 Table 4: Share of Indirect Exporting Firms and Ownership Status

| **Ownership Status of Indirect Exporting Firm** | **Yes** | **No** | **Grand Total** |
| --- | --- | --- | --- |
| Sole Proprietorship | 5.07% | 37.79% | 42.86% |
| Partnership | 6.45% | 17.05% | 23.50% |
| Private Ltd Liability Company | 18.89% | 5.99% | 24.88% |
| Public Ltd Liability Company | 4.61% | 4.15% | 8.76% |
| **Grand Total** | **35.02%** | **64.98%** | **100.00%** |

S2 Table 5: Top 8 Export Destinations

| Sr. | Export Destinations | Frequency of Exporting Firms |
| --- | --- | --- |
| 1 | USA | 87 |
| 2 | UK | 79 |
| 3 | Italy | 63 |
| 4 | Spain | 56 |
| 5 | Germany | 47 |
| 6 | France | 44 |
| 7 | Australia | 36 |
| 8 | Canada | 26 |

S2 Table 6: Variables, and their Measurement

| **Variable** | **Measurement** |
| --- | --- |
| Foreign Outsourcing Collaboration | The proportion of total Exports is linked to indirect exports i.e., exports through foreign outsourcing collaboration |
| Wage to Labor Productivity Ratio | Wage to labor-productivity is calculated by adding wages of unskilled, skilled, and higher-level management employees paid in the fiscal year 2022 and dividing by labor productivity (i.e., total output divided by total labor). |
| Skilled Labor | Total number of skilled employees having skill certification |
| Foreign Headquarter Services | Foreign Headquarter Services are the proportion of total inputs received by operators of manufacturing plants from the final goods producer. |
| Share of Cost | Share of cost is calculated by dividing the cost of sales by the total production |
| Share of Cost Squared | The share of cost squared is the square of the share of cost |
| Variety | Total number or a variety of goods produced by the firm |
| Domestic Inputs | The proportion of domestic inputs used in the production of textile and apparels related goods. |
| Distribution/ Transportation | The extent to which distribution/ transportation activity is important to achieve competitive advantages: Not Important at all [1]; Slight Important [2]; Moderate Important [3]; Very Important [4]; Extremely Important [5] |
| Marketing | The extent to which marketing activity is important to achieve competitive advantages: Not Important at all [1]; Slight Important [2]; Moderate Important [3]; Very Important [4]; Extremely Important [5] |

S2 Table 7: Descriptive Statistics

| **Variable** | **Observations** | **Mean** | **Std. Dev.** | **Min** | **Max** |
| --- | --- | --- | --- | --- | --- |
| Foreign Outsourcing Collaboration | 217 | 0.29 | 0.43 | 0.00 | 1.00 |
| Wage to Labor Productivity Ratio | 217 | 88.97 | 220.94 | 0.14 | 2104.43 |
| Skilled Labor | 217 | 1.25 | 1.82 | 0.00 | 6.43 |
| Foreign Headquarter Services | 217 | 0.53 | 0.34 | 0.00 | 1.00 |
| Share of Cost | 217 | 0.85 | 0.10 | 0.06 | 0.98 |
| Share of Cost Squared | 217 | 0.72 | 0.12 | 0.00 | 0.96 |
| Variety | 217 | 3.04 | 2.27 | 1.00 | 12.00 |
| Domestic Inputs | 217 | 0.88 | 0.19 | 0.00 | 1.00 |
| Distribution/ Transportation Activities | 217 | 3.02 | 0.88 | 1.00 | 5.00 |
| Marketing Activities | 217 | 2.91 | 1.57 | 1.00 | 5.00 |

S2 Table 8: Heteroscedasticity Test

| Test | Model 1 | | Model 2 | | Model 3 | |
| --- | --- | --- | --- | --- | --- | --- |
|  | $\chi^{2}$ | Prob | $\chi^{2}$ | Prob | $\chi^{2}$ | Prob |
| Breusch Pagan/Cook Weisberg test | 22.65 | 0.0000 | 21.18 | 0.0000 | 21.40 | 0.0000 |
| White’s test | 65.77 | 0.0009 | 68.54 | 0.0079 | 74.76 | 0.0261 |

**Note:** $\chi^{2}$ indicate chi-square.

S2 Table 9: Determinants of FOC in the Textile Sector of Pakistan

|  | **Model 1** | **Model 2** | **Model 3** |
| --- | --- | --- | --- |
|  | OLS with Robust SEs | OLS with Robust SEs | OLS with Robust SEs |
| Wage to Labor Productivity Ratio | -0.000133* | -0.000127* | -0.000128* |
|  | (0.0000701) | (0.0000705) | (0.0000716) |
|  |  |  |  |
| Skilled Labor | 0.0880*** | 0.0870*** | 0.0862*** |
|  | (0.0107) | (0.0109) | (0.0122) |
|  |  |  |  |
| Foreign Headquarter Services | 0.224*** | 0.241*** | 0.232*** |
|  | (0.0382) | (0.0384) | (0.0592) |
|  |  |  |  |
| Share of Cost | 0.953** | 1.059** | 1.047** |
|  | (0.326) | (0.344) | (0.351) |
|  |  |  |  |
| Share of Cost Squared | -0.663* | -0.774* | -0.763* |
|  | (0.333) | (0.365) | (0.370) |
|  |  |  |  |
| Variety | 0.0529*** | 0.0527*** | 0.0528*** |
|  | (0.00988) | (0.0101) | (0.0102) |
|  |  |  |  |
| Domestic Inputs | -0.155 | -0.133 | -0.133 |
|  | (0.126) | (0.100) | (0.100) |
|  |  |  |  |
| Distribution/ Transportation |  | -0.0317 | -0.0314 |
|  |  | (0.0361) | (0.0361) |
|  |  |  |  |
| Marketing |  |  | 0.00352 |
|  |  |  | (0.0112) |
|  |  |  |  |
| _cons | -0.279 | -0.220 | -0.222 |
|  | (0.178) | (0.244) | (0.244) |
| *N* | 217 | 217 | 217 |
| *R*^2^ | 0.458 | 0.462 | 0.463 |
| adj. *R*^2^ | 0.440 | 0.442 | 0.439 |

**Note:** Standard errors in parentheses; ^*^ *p* < 0.10, ^**^ *p* < 0.05, ^***^ *p* < 0.01

S2 Table 10: Test for Multicollinearity

| **Variable** | **VIF** | **1/VIF** |
| --- | --- | --- |
| Wage to Labor Productivity Ratio | 1.16 | 0.859197 |
| Skilled Labor | 1.76 | 0.568901 |
| Foreign Headquarter Services | 2.18 | 0.458075 |
| Share of Cost | 1.03 | 0.967200 |
| Variety | 1.53 | 0.653109 |
| Domestic Inputs | 1.27 | 0.790365 |
| Distribution/ Transportation | 1.07 | 0.936033 |
| Marketing | 2.15 | 0.465201 |
| Mean VIF | 1.52 |  |

S2 Table 11: Ramsey RESET Test for Omitted Variables

| Omitted: Powers of independent variables |
| --- |
| H0: Model has no omitted variables |
| F(26, 182) = 1.06 |
| Prob > F = 0.3952 |
