# Supplementary material for: Foreign outsourcing collaboration within a developing economy’s perspective: A case of the Pakistani textile industry
Source: PLoS One. 2024 Apr 16;19(4):e0299454. doi: 10.1371/journal.pone.0299454 (PMC11020694; doi:10.1371/journal.pone.0299454)
Supplement: S1 Questionnaire — (PDF) [file pone.0299454.s003.pdf]

Serial Number: ---------------------------------
Date of Interview: ------------------------------

Questionnaire No. |__|__|__|

Firm Code: |__|__|

Industrial Estate: ___

FIRM FOREIGN OUTSOURCING COLLABORATION SURVEY

Outsourcing is an in-house or ongoing activity once performed by the firm's own employees and staff and is currently performed by a party outside the company. Whereas **foreign outsourcing collaboration** is a decision made by domestic firms to collaborate with foreign firms in their foreign outsourcing strategy.

Basic Information about Survey and Consent for Filling Questionnaire

The survey is being conducted to study the foreign outsourcing collaboration (FOC) activities, agglomeration, and product & process innovation associated issues. You are invited to participate in this Ph.D. research. Please note that your involvement is completely voluntary. Once you agreed to participate, you will be interviewed using a questionnaire. Findings of the study would benefit textile and garment industry by enhancing the exports through FOC activities which helps in achieving cost efficiency and ultimately boost profit margins and enhance the competitiveness of the industry. We shall also suggest several policy recommendations for the inculcation of R&D culture and innovation activities as well as boosting the exports of the textile sector, such a policy would help in reducing barriers to international trade including reduction of tariff & non-tariff barriers and improving the ease-of-doing business.

The researcher is a Ph.D. scholar at the National University of Sciences and Technology (NUST), Islamabad and is a regular faculty member of Balochistan UET Khuzdar. We assure that information provided by you would be kept confidential and anonymous. Only accumulated outcomes based on the field survey will be used for disseminating and publishing study results. Please note that your participation is entirely voluntary and deciding not to participate will not affect your relationship to the researchers or associated organizations. Once you agreed to participate, you will be interviewed using a questionnaire. You may choose not to answer some of the questions or whole if you feel so. It will take approximately 20-25 minutes and you may of course decide to stop the interview at any point. Thank you very much.

Would you like to take part in the study? [ ] *Yes =1 No = 0 (find next Firm if answer is No)*

Date of interview:______________________
1st visit: / / 2nd visit: / / 3rd visit: / /
Name of Interviewer: _______________________________________
Name of Supervisor:____
Checked by:____
(Name & Signature)
Edited by: ____
(Name & Signature)

INSTRUCTIONS FOR ENUMERATORS

- Multiple ticks (√) are permitted; always inquire whether the respondent wishes to select more than one choice.
- Please choose the suitable option (e.g., **1= YES, and 0= NO**) and do not leave any entry blank.
- When consecutive/successive numeric entries reveal inconsistencies and do not make sense, please verify the answers, or explain the question again, if the respondent is not clear.
- *, ** indicates that explanation is given in Table Note.

PERSONAL INFORMATION OF RESPONDENTS

P11: Respondent Name		P12: Respondent Cell No.		P13: Gender	**Male [ ]; Female [ ]**
P14: Education Level	**No schooling [ ];<br>Matriculation [ ];<br>Diploma in technical/ vocational training [ ];<br>Intermediate [ ]; Bachelor's degree [ ];<br>Master's degree [ ]; Doctorate [ ].**	P15: Employment Status	**CEO [ ]<br>Director [ ]<br>Manager [ ]<br>Account Officer [ ]<br>Any other:**	P16: Work Experience	**[ ] years<br>[ ] months**

F1. Firm GPS Coordinates and Address

F1: Firm Name	F11: Firm GPS Coordinates		F12: Contact # (Firms)	F13: Approx. Factory area	F14: Address	F15: Location of the firm	F16: If answer to F15 is **[1 or 2 or 3],** Name of Industrial area or Export Processing Zone or Special Economic Zone where firm is located	F17: Location of Plant
	latitude	longitude						
				**(Marla or Kanal)**		**1, 2, 3, 4**		**Urban, Rural**

Scale: Industrial Area/Park **[1]; **Export Processing Zone **[2]; **Special Economic Zone **[3]**; None **[4]**

F2. In which industry your enterprise operates?

Division	Group	Class	Subclass	Industry Major Groups and Industries	Choose Yes, No
13				**Manufacture of textiles**	
	139			Manufacture of other textiles	
		**1392**		**Made-up textile articles, except apparel**	
			**13921**	Made-up articles of any textile material	**Yes, No**
			**13922**	Made-up furnishing articles	**Yes, No**
			**13929**	Others, n.e.c.	**Yes, No**
**14**				**Wearing Apparel**	
	**141**			**Wearing apparel, except fur apparel**	
		**1410**		Wearing apparel, except fur apparel	
			**14101**	Wearing apparel of leather or composition leather	**Yes, No**
			**14102**	Outerwear/ work wear	**Yes, No**
			**14103**	Underwear/ nightwear	**Yes, No**
			**14104**	Babies' garments/ tracksuits, etc.	**Yes, No**
			**14105 &**	Other clothing accessories such as gloves, belts, shawls, ties, cravats, hairnets etc./ hats and caps/	**Yes, No**
			**14109**	Others, n.e.c.	**Yes, No**
	**143**			**Knitted & crocheted apparel**	
		**1430**		Knitted & crocheted apparel	
			**14301**	Knitted or crocheted wearing apparel	**Yes, No**
			**14302**	Hosiery, socks, tights & pantyhose, etc.	**Yes, No**

Note: "n.e.c" stands for "not elsewhere classified".

A1. The basic structure of the Firm

A11: Year of the establishment of the Firm (فرم کے قیام کا سال)	
A12: Ownership status of your firm (آپ کی فرم کی آنرشپ اسٹٹس)	**1, 2, 3, 4**

Scale A12: Sole proprietorship [1]; Partnership [2]; Private limited Liability Company [3]; Public Limited Liability Company [4].

B1. Firm Operations

B11: Total number or a variety of goods produced by the firm. (کل تعداد یا فرم کے طرف سے تیار کردہ سامان کی اقسام۔)	**(counts)**

B2. Wages

B21: Average Wage firm pays	B211: Unskilled labor	**PKR.**
	B212: skilled labor	**PKR.**
	B213: Managers& higher- level staff	**PKR.**

B3. Employment

B31: Total Number of Employees (ملازمین کی کل تعداد)	
B32: Total number of skilled employees (having skill certification) (ہنر مند ملازمین کی کل تعداد (جن کے پاس اسکل سرٹیفکیٹ ہو)	

B4. Firm Exports

B71: Does your firm operate as a direct exporting firm? (کیا آپ کی فرم براہ راست برآمد کرنے والی فرم کے طور پر کام کرتی ہے؟)	**Yes, No**
B41: Does your firm operate as an indirect exporter through foreign outsourcing collaboration? (کیا آپ کی فرم فارن آؤٹسورسنگ کولابریشن کے ذریعے بالواسطہ برآمد کنندہ کے طور پر کام کرتی ہے؟)	**Yes, No**
B42: What %age of total Exports is linked to indirect exports (i.e., exports through foreign outsourcing collaboration) (کل برآمدات کی کتنی فیصد بالواسطہ برآمدات سے منسلک ہے۔)	**%**

B5. Local Business Activity

**B51:** Does your company use local raw materials? (کیا آپ کی کمپنی مقامی خام مال استعمال کرتی ہے؟)	**Yes, No**

B6. Cost of Production/Production (PKR million)

B61: Cost of sales (C)	**PKR.**
B62: Total Sales Value or Total Production (Q)	**PKR.**

B7. Foreign Headquarter Services

B71: Does your firm receive the following inputs from foreign outsourcing collaborating firm (final goods producers)? (کیا آپ کی فرم کو فارن آؤٹسورسنگ کولابریشن کرنے والی فرم (فائنل گڈز پروڈیوسرز) سے درج ذیل ان پٹ موصول ہوتے ہیں؟)	B711: design specifications	**Yes, No**	B716: Poly Bags	**Yes, No**
	B712: quality requirements	**Yes, No**	B717: Stickers	**Yes, No**
	B713: Dimensions/size	**Yes, No**	B718: Tags	**Yes, No**
	B714: Yarn	**Yes, No**	B719: Labels	**Yes, No**
	B715: Insert Cards	**Yes, No**	B71x: Any other (please specify)	**Yes, No**

D1. Foreign and Domestic Outsourcing Collaborations

D11. Types of outsourcing collaborations your enterprise is indulged in	**1, 2, 3, 4**

D16: If **"Foreign Outsourcing Collaboration"** is chosen for **D11**, in which countries firm's foreign outsourcing collaboration partners operates (choose name of countries or write names if not listed)

D161: China	D164: UK	D167: Sri Lanka	D16x: Spain	D16x3: Germany	D16x6:
D162: Japan	D165: Netherlands	D168: Sweden	D16x1: Finland	D16x4: Italy	D16x7:
D163: USA	D166: Bangladesh	D169: Belgium	D16x2: Australia	D16x5:	D16x8:

Scale D11: Foreign Outsourcing Collaboration [1], Domestic Outsourcing Collaboration [2], Both [3], None [4].

B8. Core competencies-- To what extent each of the following activity is important to achieve competitive advantages in your industry
(آپ کی صنعت میں کمپیٹیٹو ایڈوانٹیج حاصل کرنے کے لیے درج ذیل میں سے ہر ایک سرگرمی کس حد تک اہم ہے۔)

B81: Distribution/ transportation	**1, 2, 3, 4, 5**
B82: Marketing	**1, 2, 3, 4, 5**

Scale: Not Important at all [1]; Slight Important [2]; Moderate Important [3]; Very Important [4]; Extremely Important [5]
